# A roadmap to pulmonary delivery strategies for the treatment of infectious lung diseases

**DOI:** 10.1186/s12951-022-01307-x

**Published:** 2022-03-03

**Authors:** Siqin He, Jiajia Gui, Kun Xiong, Meiwan Chen, Huile Gao, Yao Fu

**Affiliations:** 1 Key Laboratory of Drug-Targeting and Drug Delivery System of the Education Ministry and Sichuan Province, Sichuan Engineering Laboratory for Plant-Sourced Drug and Sichuan Research Center for Drug Precision Industrial Technology, West China School of Pharmacy, Sichuan University, Chengdu, 610041 China; 2 State Key Laboratory of Quality Research in Chinese Medicine, Institute of Chinese Medical Sciences, University of Macau, Macau, China

**Keywords:** Drug delivery, Lung, Pulmonary infectious disease, Targeting, Therapeutic strategies

## Abstract

Pulmonary drug delivery is a highly attractive topic for the treatment of infectious lung diseases. Drug delivery via the pulmonary route offers unique advantages of no first-pass effect and high bioavailability, which provides an important means to deliver therapeutics directly to lung lesions. Starting from the structural characteristics of the lungs and the biological barriers for achieving efficient delivery, we aim to review literatures in the past decade regarding the pulmonary delivery strategies used to treat infectious lung diseases. Hopefully, this review article offers new insights into the future development of therapeutic strategies against pulmonary infectious diseases from a delivery point of view.

**Graphical Abstract:**

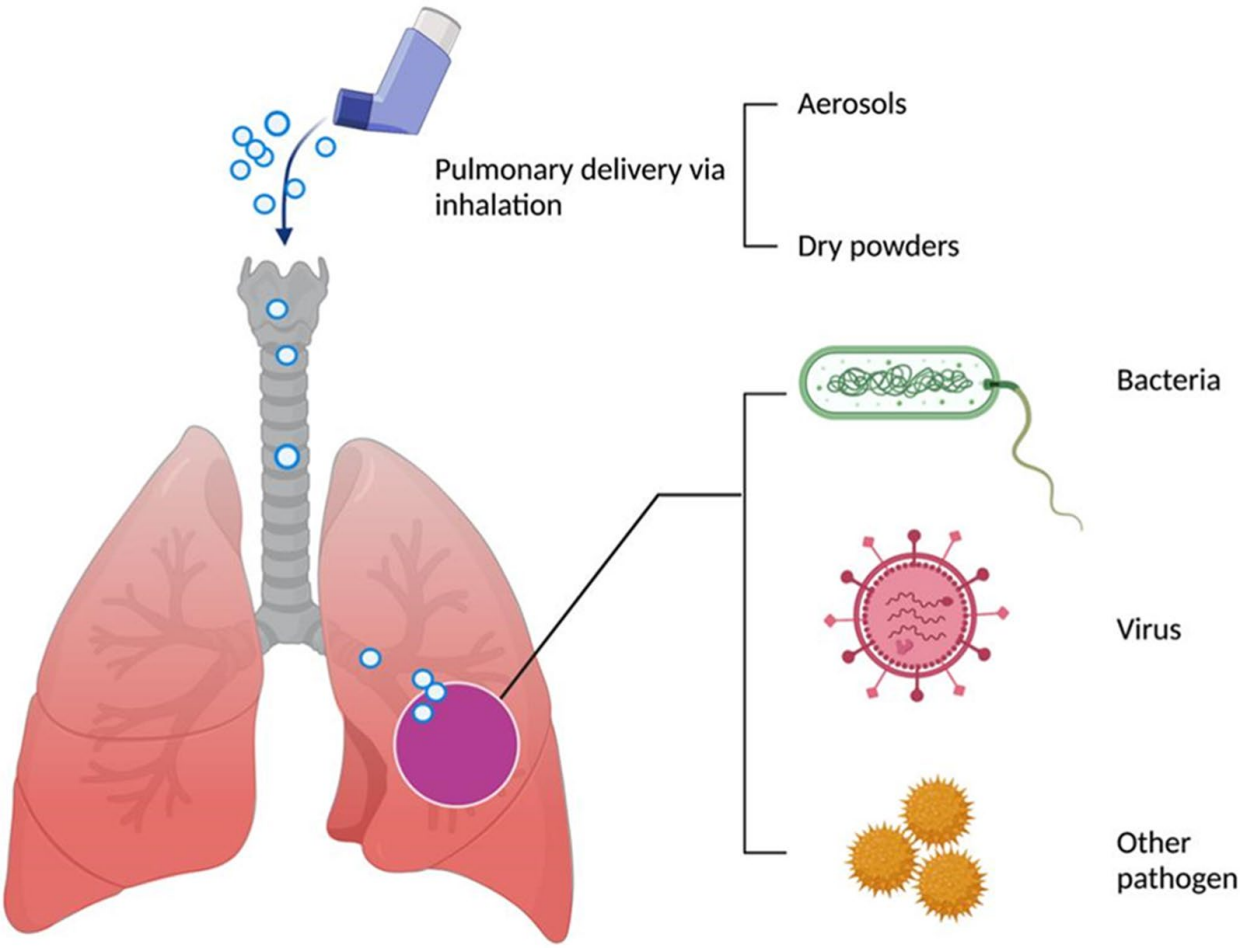

## Introduction

The ongoing pandemic caused by SARS-CoV-2 has swept into at least 223 countries worldwide with more than 5,301,714 people killed as of Dec. 14, 2021, according to statistics by the World Health Organization (WHO). Unfortunately, the numbers are continuously growing with the appearance of coronavirus variants such as the Delta strain first discovered in India, October 2020. The SARS-CoV-2 viral infection attacks the lung which results in a lack of oxygen and systemic inflammation causing acute respiratory distress syndrome and damages vital organs such as kidney [1], liver [2], heart [3], and brain [4].

Pulmonary infectious diseases have long been a major category of diseases severely threatening human life and global health. WHO statistics reveal that pneumonia has been the leading cause of death among children, and in 2017, an estimated number of 808,694 children died of pneumonia under the age of five globally, accounting for 15% of the mortality in children under five. In addition, tuberculosis (TB) remains one of the top 10 causes of death in the world population, about 1.6 million people died of TB infection in 2017 and over 10 million people currently live with TB [4]. Overall, the global impact of pulmonary infectious diseases is tremendous, causing extensive social and economic burdens to society. However, great challenges remain for finding effective yet safe treatment options against pulmonary infectious diseases. For the development of antiviral therapeutics against SARS-Cov-2, besides vaccines, some drugs have entered the clinical stage and have shown good antiviral potential. REGEN-COV is a cocktail therapy consisting of two monoclonal antibodies (casirivimab and imdevimab) that target the receptor binding domain (RBD) on the spike protein of SARS-CoV-2 to prevent the virus entry into the host cell. REGEN-COV shortens the duration of symptoms and reduces the patient's risk of death, and REGEN-COV treatment in humans will not lead to virus mutations [5]. Sotrovimab is an engineered human monoclonal antibody, which directly targets the spike protein of SARS-CoV-2 to block its attachment and entry into human cells. In patients with mild to moderate Covid-19 infection, sotrovimab reduces the risk of disease progression [6]. Remdesivir is the first officially approved small molecule drug for the treatment of COVID-19 in the United States. Remdesivir is a nucleoside analog prodrug that has successfully improved the recovery rate of moderate and severe Covid-19 patients in the hospital, however, remdesivir treatment is shown to be less associated with patient survival [7, 8]. Molnupiravir is an oral antiviral drug recently approved by FDA on an emergency basis, which can be used for the treatment of mild to moderate COVID-19 adult patients [9]. Molnupiravir works on viruses by increasing the mutation rate in the viral genome to interrupt the virus replication and spread. PF-07321332 is an oral 3CL protease inhibitor, which plays an important role in the life cycle of a variety of coronaviruses and has extensive antiviral activity against human coronavirus in vitro. Due to the rapid mutation of the virus, the research and development of anti-SARS-CoV2 drugs has a long way to go [10]. On the other hand, some potential therapeutic compounds failed in clinical trials due to problems such as insufficient therapeutic concentrations at the target site and adverse effects resulting from off-target distributions. Chloroquine, for example, has initially been proposed as a candidate drug for SARS-CoV-2 infection, which is proven to inhibit pH-dependent viral replication and downregulate the levels of IL-6 and TNF-α through immune regulation in vitro [11]. However, it has been shown to cause serious adverse effects such as cardiotoxicity, resulting in ventricular arrhythmia, conduction block, and cardiovascular failure [12]. Considering that most elderly patients or patients with cardiovascular complications are targets of SARS-CoV-2, inappropriate use of chloroquine may increase the risk of heart-related death [13]. Also, the therapeutic window of chloroquine is extremely narrow, and as a result, even low-dose or medium-dose chloroquine would cause myocardial dysfunction, which remains difficult to control the effective concentration of chloroquine in the safe range [14]. For this type of therapeutics, it is important to develop drug delivery systems (DDS) to enhance site-specific drug accumulation or to achieve precise delivery towards the disease site.

Among various delivery systems or strategies, pulmonary drug delivery offers unique advantages such as no first-pass effect and high bioavailability, which can deliver therapeutics directly to lung lesions. With the ongoing pandemic, there is an emerging body of evidence that numerous antiviral pulmonary inhalations are currently under clinical studies. Hence, this review article mainly focuses on the physiological structure of the lung, the causes of pulmonary infections, and pathological changes, to explore pulmonary drug delivery strategies for the treatment of infectious lung diseases. Specifically, localized delivery systems for pulmonary administrations will be discussed to provide insights into the future development of therapies for pulmonary infectious diseases and for seeking effective treatment alternatives.

## Lung anatomy, functions and barriers for pulmonary drug delivery

### Anatomy and biological functions of lung

**Fig. 1:**
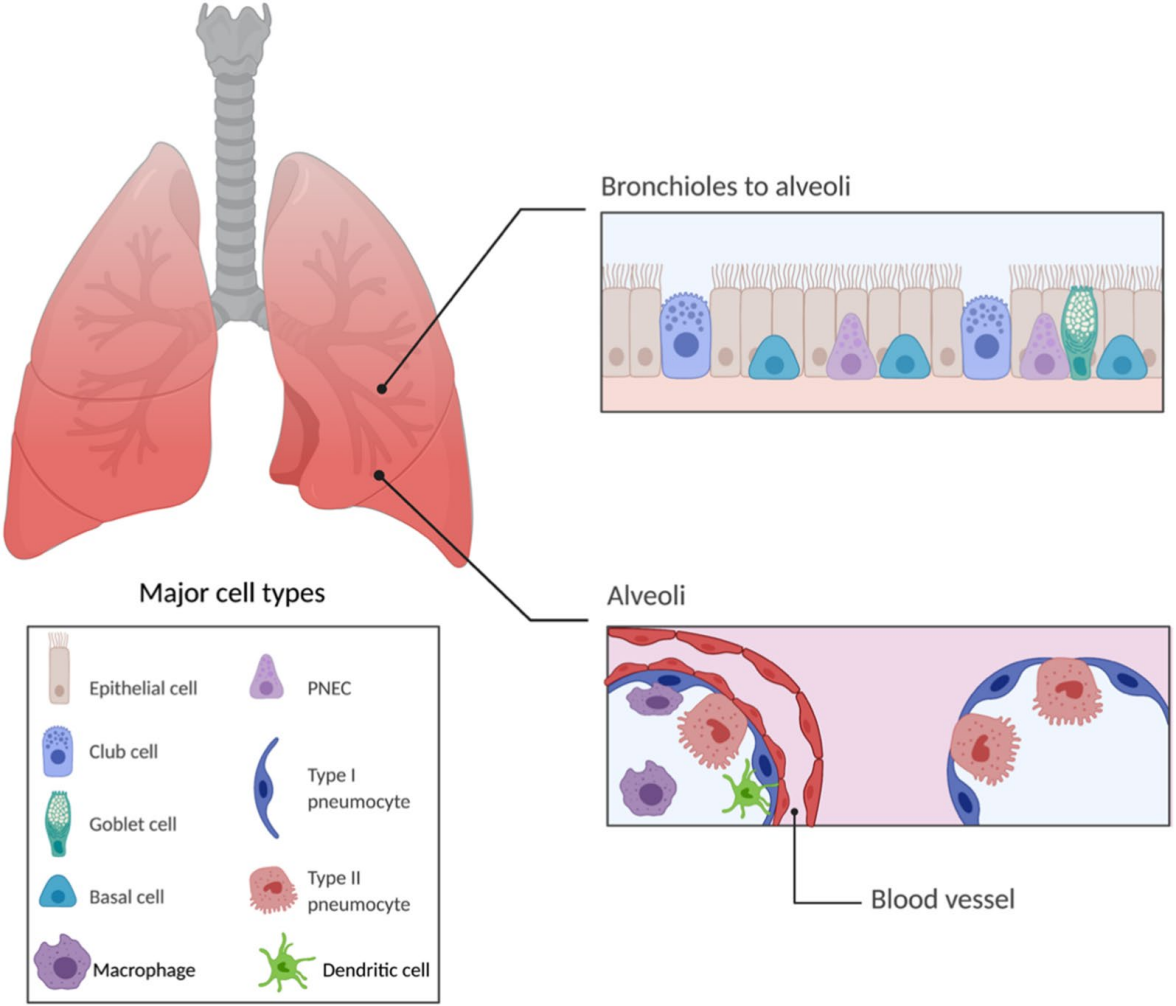
Schematic drawings of the lung anatomy and major cell types. The airway epithelium is a continuous cell layer that mainly consists of epithelial cells (ciliary cells), basal cells, goblet cells, club cells, and pulmonary neuroendocrine cells (PNEC). Alveoli are the smallest passageway in the respiratory system, which consist of type I and type II pneumocytes. Type 1 pneumocytes allow gas exchange, while type II pneumocytes are responsible for secreting surfactants. Alveolar macrophages (AMs) and dendritic cells are responsible for clearing up debris through phagocytosis [16, 17] (Figure created in BioRender.com)

As the respiratory organ of the human body, the lung is composed of the terminal airway, alveoli and pulmonary interstitium, which can be further divided into two main regions: the conductive airway region and the respiratory region. The structure of the lung determines its ventilation function. The conductive airway consists of the nose, pharynx, larynx, trachea, bronchial tube, bronchioles, and terminal bronchioles, while the respiratory region includes respiratory bronchioles, alveolar ducts, and alveolar sacs [15]. A connective tissue stroma exists between bronchi and alveoli, in which lymphatic vessels, nerves, and blood vessels are also distributed, as well as macrophages, fibroblasts, and other immune cells (Fig. 1) [16, 17].

The lungs contain more than 300 million alveoli consisting of type I and type II pneumocytes (Fig. 1). A rich capillary network in the interstitium exists between the alveoli, which has a surface area of nearly 70 m [2] forming a blood-gas barrier for gas exchange between the blood and alveoli. When oxygen travels from alveoli to blood, it needs to pass through the respiratory membrane composed of the alveolar wall and blood vessel wall with a thickness of about 0.5–1.0 µm [18]. This is also the case for drug absorption following pulmonary route of administration. The respiratory membrane is highly permeable with sufficient blood flow in the lungs, which can avoid the first-pass effect following systemic administration. Thus, pulmonary drug delivery represents a promising route of drug administration especially for therapeutic drugs that easily undergo liver metabolism following the first-pass effect. Also, localized delivery offers a direct approach to achieve enhanced drug accumulation at the lung, which will likely benefit pulmonary disease treatment.

### Biological barriers for pulmonary drug delivery

**Fig. 2:**
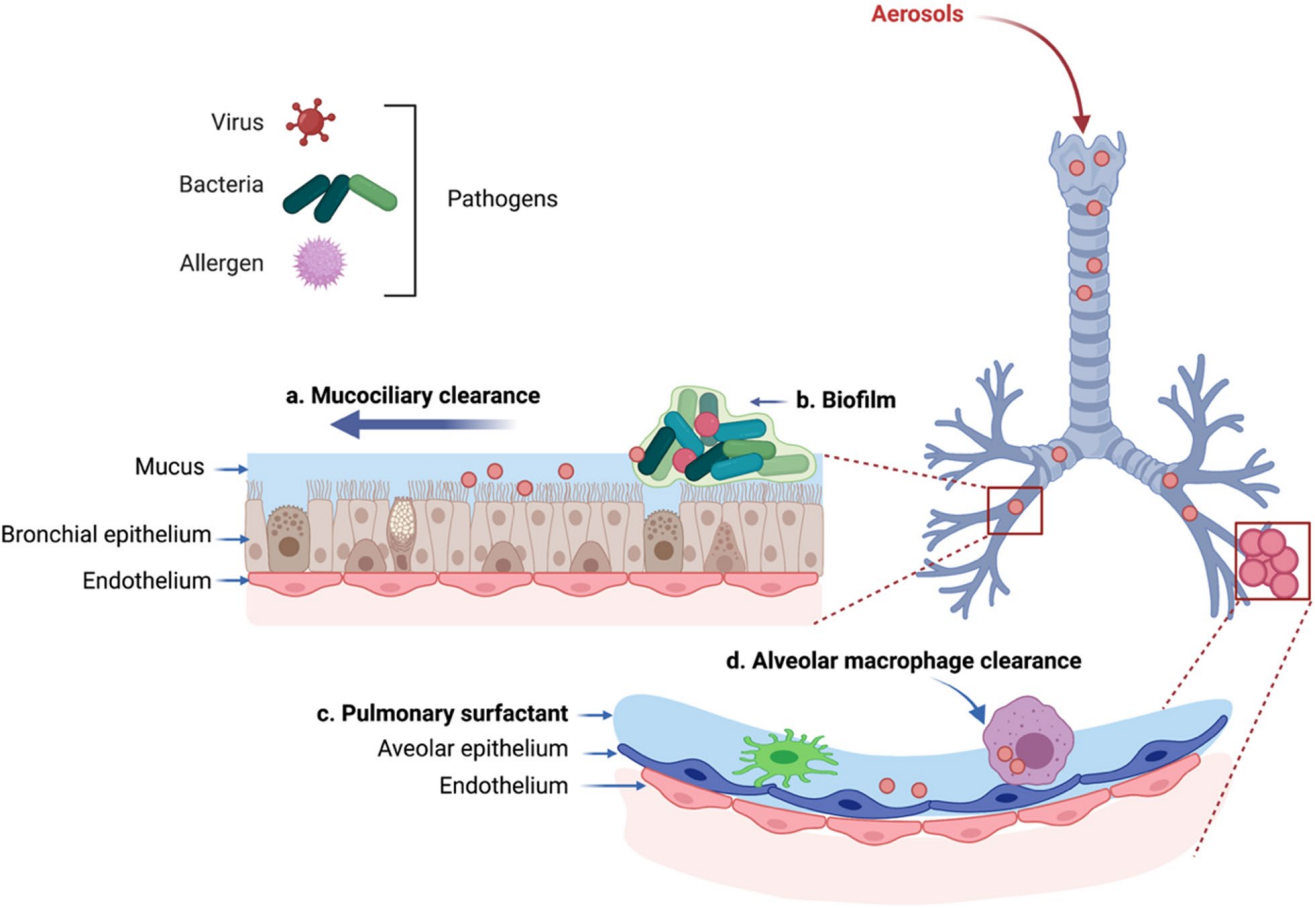
The biological barriers for pulmonary drug delivery towards lung infections. **a** Mucociliary clearance, **b** biofilm, **c** pulmonary surfactant and **d** alveolar macrophage clearance. Adapted from Ref. [16] (Figure created in BioRender.com)

Regarding pulmonary inhalation, drugs delivered via inhalation are exposed to multiple clearance mechanisms, which constitute the main barriers to drug absorption following pulmonary administration (Fig. 2).

#### Mucociliary barrier

Mucociliary clearance (Fig. 2a) is one of the most important defense mechanisms of the lung, which helps remove inhaled particles and exogenous substances [16]. For example, dusts and microbes from the back of the nasal cavity, or the throat to the terminal bronchioles, as well as drugs that cannot pass through the mucus layer or the lung epithelial cells are removed via mucociliary clearance [19].

The mucociliary clearance system consists of the periciliary layer (PCL) and mucous layer. The cilia are located on the top surface of the airway epithelial cells, and the mucous layer is located on the top of the ciliated layer [20]. PCL is a water-rich layer occupied by graft brushes of transmembrane mucins and mucopolysaccharides [21]. Inhaled drugs are easily intercepted by viscoelastic mucus and PCL making the cilia move regularly, pushing exogenous particles along with the mucus toward the pharynx so that they are eventually expelled from the respiratory tract. The mucus layer is composed of mucin MUC5AC and mucin MUC5B [22, 23]. Mucin glycoproteins are the main functional component of the mucus [24]. Mucin monomers are connected by cysteine bridges in the aqueous media to form mucin fibre networks with distances ranging from 50 to 1800 nm [19, 20], which can intercept drug particles in a certain range of particle size. After inhalation, the drug molecules or particles are trapped in the mucous layer, and the mucus barrier affects the dissolution of the drug, hinders the epithelial diffusion of the drug, and the interaction of the drug with the cell surface and receptors [25]. Thereby, how to overcome the mucus layer to reach the alveolar epithelial layer remains a critical biological barrier for efficient drug delivery.

The size and surface properties of particles may impact the interaction between the particles and the mucus layer thus the efficiency of the delivery system. The size of the particle should be well modulated to allow the particles to pass through the mucin without being affected by the mucus rheology [26, 27]. Regarding charge properties, the mucus is negatively charged, and the negative surface charge of the particle has been proven to benefit mucus penetration, but a repulsion effect does exist. Although positively charged particles are more likely to interact with negatively charged epithelial membranes and trigger endocytosis, they may easily adsorb mucus proteins which become difficult to penetrate the mucus layer. Moreover, studies have shown that neutral nanoparticles (NPs) spread faster through the mucus layer because their neutral surface charge tends to reduce hydrophobic and electrostatic interactions [28].

#### Pulmonary surfactant

Pulmonary surfactant (PS) (Fig. 2b) is a lipoprotein complex with amphiphilicity, which is secreted by alveolar type II (AT-II) cells in the pulmonary epithelium. PS consists of phospholipids and surfactant protein [28]. The surfactant proteins can be divided into four types: SP-A, SP-B, SP-C, and SP-D. SP-A and SP-D can promote the adhesion and agglutination of certain drugs by mucosal cilia or macrophages and monocytes, and jointly participate in the innate defense of alveolar by improving the pathogen clearance rate and the specific regulation of inflammatory immune response. Lung surfactants can also be involved in the removal of therapeutic drugs or drug carriers [29].

#### Immune cells

Pulmonary macrophages (Fig. 2c) and dendritic cells, which endocytose and eliminate apoptotic cells and foreign bodies, form the first line of defense, especially in the alveolar region. Macrophages first recognize exogenous substances, swallow them and then migrate to the respiratory tract, and remove the substances from the body under the action of mucus. The endocytosis is the main reason that hinders the absorption of some lung inhalation drugs, especially macromolecular biologics [30], and is related to the size of exogenous substances, which tends to internalize particles ranging from 0.5 to 5.0 μm [16, 31].

In addition to phagocytosis, macrophages play an important role in the occurrence, development and physiological processes of various lung infectious diseases. Macrophages participate in wound healing and maintain tissue stability by changing their phenotypes, which is proven critical in mediating an effective immune response against pathogens [32]. Macrophage polarization generates two subtypes: proinflammatory macrophages (M1-like), and anti-inflammatory macrophages (M2-like) [33]. The M1/M2 macrophage polarization is a tightly coordinated process. In the early stage of infection, macrophages polarize towards the M1 phenotype, leading to the production of massive inflammatory factors such as reactive nitrogen intermediates and reactive oxygen species, which kill pathogens and triggers adaptive immunity. To balance excessive inflammation, macrophages then undergo apoptosis or differentiate into M2 phenotype to protect the host from injury and trigger the healing process. However, certain pathogenic microorganisms, such as mucosa streptococcus, promote the occurrence of inflammatory reactions, causing unbalanced M1/M2 polarization, and eventually leading to severe diseases [32, 34, 35]. In response to this situation, the most common strategy is to increase M2-like or to reduce M1-like phenotype, such as the use of the drug regorafenib [36, 37]. Hence, targeting specific macrophage phenotype may provide alternative treatment options for pulmonary infectious diseases.

#### Enzymes

Despite the above biological barriers, the metabolic enzymes in lung epithelial cells maintain another barrier function. Metabolic enzymes such as antitrypsin, protease, and trypsin, are found on the surface of bronchial and alveolar epithelial cells and pulmonary smooth muscle cells, which contribute to the degradation and the metabolism of therapeutic drugs [38].

#### Biofilm

Although biofilm is not part of the lung tissue, biofilm presents a major physical barrier for pulmonary inhalation. Biofilm is a structured community formed by microbial cells surrounded by their extracellular matrix and attached to the inert or biological surface (Fig. 2d) [39], which is composed of polysaccharides, extracellular polymer, lipid, and DNA [40], functioning as a major barrier to the efficient penetration of antimicrobial agents. Some bacteria have a strong capacity to form biofilm s, such as epidermal *Staphylococcus aureus* [41], and *copper-green pseudomonas* [42], which adhere to the mucous layer and to each other. Once the biofilm forms, bacteria become resistant to antimicrobials and the body’s immune removal. For example, tobramycin, positively charged antibiotics, may interact with biofilm components, resulting in a decreased efficacy due to slow and incomplete drug penetration into the biofilm matrix [43]. The low pH in the biofilm causes alkaline drugs to protonize through the charge interaction to enhance the interaction with alginate in the biofilm, thereby reducing the concentration of free drugs at the site of the action [44].

## Pulmonary infectious diseases

Pulmonary infections are induced by various pathogenic microorganisms resulting in varying degrees of lung injury. Inflammation functions as a major defensive response to pathogen-induced tissue injury. The general process of inflammation includes metamorphism, exudation, and proliferation, corresponding to the initiation of inflammation, tissue injury, and repair, involving multiple cellular pathway changes and signal transduction processes [45]. However, inflammation is also a double-edged sword, for example, uncontrolled cell activation, the release of cytokines, and chemokines may lead to fatal “cytokine storms” that damage their tissues and cells. In severe cases, it can lead to fatal symptoms such as pulmonary fibrosis, acute respiratory distress syndrome and even respiratory failure [46, 47].

Bacteria pneumonia is caused by *Streptococcus pneumoniae* [48], *Klebsiella pneumoniae* [49], *Staphylococcus aureus* [50], and *Haemophilus influenzae* [51]. Some of the pathogenic bacteria are extracellular bacteria, which do not enter the cells, but mainly locate in the tissue fluid, blood, lymphocytic fluid, and other extracellular fluid, where they could ingest nutrients for growth and reproduction [52]. Common extracellular bacteria include gram-positive bacteria such as *Staphylococcus aureus* [53] and *Streptococcus* [54], and most gram-negative bacteria such as *copper-green pseudomonas* [55], and *Haemophilus influenza* [56]. The pathogenic mechanisms are related to the external toxins secreted by gram-positive bacteria and the lipopolysaccharide (LPS) released by gram-negative bacteria. LPS is a major component of the cell wall from gram-negative bacteria, which consists of lipids and polysaccharides. Direct exposure of LPS to the lungs not only triggers pulmonary inflammation but also promotes oxidative stress significantly, followed by activation of neutrophils and macrophages [57].

Intracellular bacteria, such as *Mycobacterium tuberculosis* (Mtb) and *Legionella*, resist the killing effect of phagocytes in different ways, survive and reproduce in phagocytes. Mtb enters the alveoli through the respiratory tract, which can be phagocytosed by macrophages and escape the destruction of intracellular pathogens by destroying the maturation of phagosomes and fusion with lysosomes [58]. Mtb then survives and reproduces within the cells, making phagocytes disintegrate and release bacteria. Currently, the first-line anti-TB drugs are isoniazid and rifampicin [59]. Most formulations are oral tablets and injections, and the treatment of TB generally lasts 6 months [60]. However, anti-Mtb treatment lasting for a long period often results in drug resistance. Currently, the aforementioned lung bacterial infections are mainly treated with antibiotics, and different types of antibiotics are used for different bacterial infections. Oral or intravenous administration is preferred [61, 62], however, the systemic delivery of antibiotics has potential issues, especially for chronic infections. High-dose antibiotics often lead to adverse reactions, resistance, and limited therapeutic accumulation at the disease site [63].

Viral pneumonia is caused by viruses including coronavirus, adenovirus, respiratory syncytial virus, influenza virus, rhinovirus, and measles virus. Viruses infect the upper respiratory tract, which then infect the lungs and invade the lining cells of the trachea, alveoli, or parenchyma. The mechanisms by which viruses infect cells and cause inflammation are diverse and complex. Recent studies revealed that SARS-CoV-2 differs from SARS-CoV by using an enzyme (TMPRSS2) to infect host cells [64]. Specifically, TMPRSS2 was found to cut the spike protein and expose the hydrophobic domain to attach to the cell membrane, whereas SARS-CoV enter cells via endosomal pathway which involves cathepsin L. The life cycle of SARS-CoV-2 viruses are summarized into the following stages: (i) viral entry, the spike protein binds to the angiotensin-converting enzyme 2 (ACE2) receptor on the host cells, i.e., lung epithelial cells; (ii) viral RNA translation inside the cell; (iii) remodelling the cell, virus transforms the endoplasmic reticulum to facilitate viral RNA replication and translation intracellularly; (iv) newly synthesized virus exit the host cell via Golgi apparatus/lysosomes. For SARS-CoV, the initial inflammatory response occurs in the early-stage after viral infection, which is mainly driven by active virus replication, down-regulation, and shedding of ACE2 mediated by envelope spike protein and host antiviral response, further leading to the production of cytokines/chemokines and cell damage caused by apoptosis [65]. In the late stage, the secondary inflammatory response begins with the production of adaptive immunity and the emergence of neutralizing antibodies, which further reduces viral replication. However, Fc receptors (FcR) interact with the viral anti-S protein-neutralizing antibody complexes, which can cause inflammation in the lungs and continuous replication of the virus, resulting in severe lung damages [66]. Therefore, blocking the FcR receptor to prevent the virus-NAb complex from binding to FcR represents a strategy to stop inflammation. Also, MERS-CoV causes even more severe symptoms with viral infections. Studies have shown that angiotensin ACE2 is the cellular receptor of MERS-CoV [67]. In the acute phase of MERS-CoV infection, severe viremia occurs, causing MERS-CoV viruses to spread in the blood circulation, which not only moisten the lungs but also cause damages to the kidney and heart. Once the virus particles enter the alveoli, AMs can no longer inhibit the spread of infection, while a strong cellular immune response may lead to inflammation and pulmonary effusion [68].

In addition to common bacteria and viruses, fungi and atypical pathogens such as mycoplasma [69], chlamydia, and legionella also lead to serious pulmonary infections [70]. Fungal pneumonia is rare, but it is more common in patients with acquired immunodeficiency syndrome, or patients taking immunosuppressive drugs that can weaken their immune systems.

## Pulmonary drug delivery

### Local inhalation

Local inhalation delivers drugs directly to the lungs to induce local or systemic therapeutic effect. Compared to other routes of administration, local inhalation has the following advantages: (i) large absorption area, thin alveolar epithelial cell membrane, high permeability, and rapid absorption; (ii) low enzyme activity, avoiding the liver first-pass effect; (iii) maintaining high concentrations of therapeutics at the target site. Hence, pulmonary inhalation has become an alternative to injections, realizing non-invasive drug administration while achieving dose reduction and increased bioavailability [71]. However, the defense mechanism evolved in the airway keeps exogenous substances out of the lung, removes or inactivates drugs after deposition [72]. Therefore, the deposition and clearance of inhaled therapeutic agents in the respiratory tract is considered one of the most important prerequisites to produce local or systemic therapeutic effects. The properties of formulations e.g., particle size, dispersity, shape, type and hygroscopicity, and the way that these formulations are administered have been the major contributing factors affecting the safety and efficiency of pulmonary drug delivery [73].

### The deposition of inhaled particles

The deposition of inhaled particles is a function of aerodynamics which refers to the average probability of particles being deposited in the airway [74]. The deposition behaviour of inhaled particles in the lung depends on the particulate properties, the individual breathing pattern, and the geometry of the airway [75–77]. Although the location of drug deposition in the lungs, and the different areas of the lung may affect the efficacy of the therapeutic drug, it remains unclear the optimum location of therapeutics deposited in the lungs, depending on the type of diseases [77].

Carvalho et al. provided an in-depth review on how the particle size influences the regional lung deposition and summarized the deposition mechanisms into inertial impaction, gravity sedimentation, and diffusion [78]. Overall, major factors that influence the bioavailability of inhalations are particle size, and their surface properties that can avoid fast absorption or deterioration in the airway. The particle size is often expressed by the mass median aerodynamic diameter, which is defined as the diameter of a sphere with a unit density of 1 g/cm^3^ and the same settling velocity as the particle of interest (Eq. 1):

1 $$ d_{aer} = d_{50} \sqrt {\left( {\frac{{\rho_{t} }}{{\rho_{a} X}}} \right)} , $$

where *d*_*aer*_ represents the aerodynamic diameter, *d*_50_ is the cutoff particle diameter, $\rho_{t}$ and $\rho_{a}$ represent the density of the particle and the density of air, respectively, and *x* indicates the shape factor ranging from 1.1 to 1.75 mm [79].

Studies have shown that the site of lung deposition corresponds to the particle size which varies dramatically due to different experimental settings [80–82]. Large particles (diameter > 5 μm) tend to deposit in the upper respiratory tract such as mouth, trachea, and main bronchi because of their relatively small inertia. Particles between 1 and 5 μm in diameter deposit in the middle end and the distal end by gravity deposition, which is considered the optimal aerodynamic diameter. However, these particles may be rapidly eliminated by macrophages in alveoli. Hence, strategies that help particles escape cellular uptake by macrophages may increase the bioavailability of the pulmonary formulation, e.g., fabrication of large porous microparticles with a low density of ~ 0.1 g/L or less [83]. Under certain circumstances, i.e., in the case of intracellular TB, AMs are the target cells, and macrophage-specific uptake is then a favoured property [84]. Aerosol particles of less than 1 μm in diameter could reach the alveoli but may often be exhaled through breathing. Ultrafine particles deposit in the airway by Brownian motion: particles < 100 nm enter the alveolar area, while particles with diameter < 10 nm deposit in the trachea-bronchial area due to a large diffusion coefficient [59, 85–87]. With appropriately designed inhalation devices such as pressurized metered-dose inhalers (pMDIs), dry powder inhalers (DPIs), and nebulizers, NPs demonstrate better penetration efficiency which can penetrate deep lung tissues and reach the alveolar area [84]. Meanwhile, cautions need to be taken to ensure that NPs reach the deep lung area without being exhaled, and moreover, the potential toxicity of NPs via inhalation remains to be carefully evaluated [88].

The deposition of drug-loaded particles in the lung correlates with their therapeutic performances. Both in vitro and in vivo models have been established to gain insights into the respiratory tract deposition of atomized drugs. In vitro simulated bronchial trees were used to observe the deposition of particles in different parts, and radioisotope-labeled aerosol particles or pharmacokinetic techniques were used to study particle deposition in vivo [78, 89]. By monitoring and comparing the concentration of inhaled and exhaled particles, the aerosol deposition in the human respiratory tract can be measured directly. Due to the limitation of the experiment, the local dose of the respiratory system remains difficult to be determined. However, studies have shown that mathematical models can be used to predict the pulmonary deposition of particles [90–93]. Different models were established to simulate the physiological environment of the human airway for evaluation. Winkler-Heil et al*.* applies aerosol dynamics model ADIC (aerosol dynamics in containments) to stochastic pulmonary deposition model IDEAL (inhalation, deposition, and exhalation of aerosols in the lungs) to forecast hygroscopic growth curves with different original particle diameters, calculate general, regional, and generational sedimentation models, and compare the calculated results of respirable particles with existing experimental data and other hygroscopic particle deposition models [94]. As a substitute for human lungs when assessing inhalation risk, the airway branches of mouse lungs were significantly asymmetric or unipolar as compared to human lungs [91]. A random and asymmetric particle deposition model was then established for Balb/c mice. Compared with the predicted deposition patterns with those in human lungs, the airway diameter is proven a more suitable morphological parameter to infer the deposition of particles in the lungs of mice and human [91].

### Inhalation formulation and devices

**Fig. 3:**
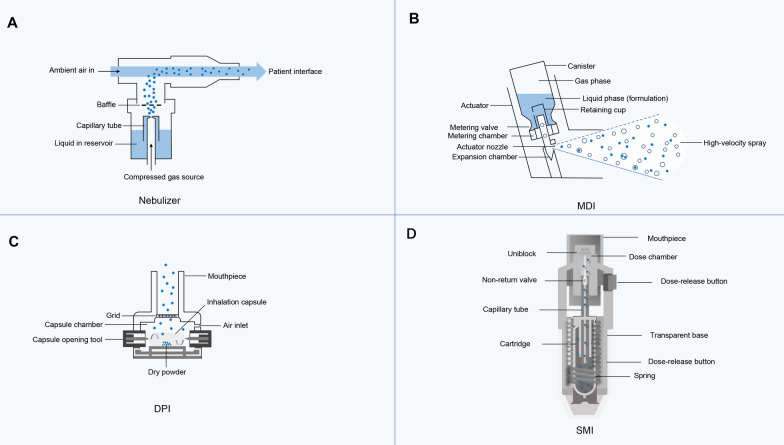
Design and operation of representative inhalation devices. The internal principle of a typical jet atomizer (**A**), a schematic diagram of a pMDI (**B**) is adapted from Ref. [96], copyright 2010, Lect Notes Eng Comp; a single-dose capsule DPI (**C**) is adapted from Ref [97], copyright 2021, Pharmaceutics; the schematic drawing of a SMI device structure (**D**). Reproduced under the terms and conditions of the Creative Commons Attribution (CCBY) license (http://creativecommons.org/licenses/by/4.0/) from Iwanaga et al. (Ref. [98]) (Figures are adapted from Refs. [95–98])

From a pharmaceutical point of view, inhalation formulations administered via the pulmonary route include nebulizers, soft mist inhalers, pMDIs, and DPIs. Several typical inhalation devices have been summarized in Fig. 3. The compressed air passes through the narrow and constricted pipe so that the 2–4 mL volume of liquid in the reservoir is sucked into the capillary and fragmented into droplets. The smaller droplets are delivered directly to the patient, and the larger droplets hit the baffle structure located near the nozzle and return to the reservoir to be atomized again (Fig. 3A) [95]. pMDI is an aerosol device for quantitative delivery of drugs to the lungs, which consists of three parts: a metal container, a metering valve, and a nozzle (or mouthpiece). Metal container is filled with prescription preparations (e.g., drugs, propellants and excipients). Metering valve is used to ensure a stable spray dose. The nozzle part of pMDI allows the drug aerosol to be inhaled into the respiratory tract (Fig. 3B) [96]. The drug capsule is placed at the bottom of the device. After being pierced by the needle, the capsule rotates with the inhaled airflow to release the drug particles contained in it (Fig. 3C) [97]. The soft mist inhalation device is a unique inhalation preparation. The main technical principle is that the mechanical energy generated by the compression spring of the rotating base provides the energy required to form and release the drug aerosol as the power, and it is accurately quantified through the capillary tube. The two medical liquid jets collide at a specific angle to form a unique soft mist through a unique collision principle (Fig. 3D) [98].

Regarding the contents loaded in various devices, they are primarily in the forms of solutions, emulsions, suspensions, and dry powders. The deposition of aerosol in the lung is affected by the aerosol generation system, particle size, drug properties, and breathing mode. Dolovich and Dhand present a systematic review on the device design and clinical use of various inhalation systems [99]. Currently, a majority of inhaled formulations are administered by atomizers, nebulizers, pMDIs, or DPIs. Among them, the passive device such as DPIs obtains the energy needed to form the aerosol from the inhaled airflow, that is, from the patient, while the active device such as pMDI is independent of the patient's inhalation to produce an aerosol. New devices have recently emerged, such as hand-held portable soft mist inhalers that do not require an external power supply and are driven by a mechanical spring [100]. Despite advances in the inhaler device design, focuses have been placed on the development of aerosol dispersion and the generation of particles with fine sizes to achieve deep lung targeting.

In the inhalation system, the atomizer converts the liquid into an aerosol. During the inhalation process, the atmospheric air passes through the atomizer for atomization transport, while in the exhalation process, the air inside the aerosol expels the aerosol to the outside of the atmosphere. Therefore, there may be residual drug leakage in the atomizer under atmospheric conditions [101]. In clinical studies, about 75% of inhaled protein formulations are prepared as spray liquids [102], possibly because atomizers have a greater advantage in maintaining protein activity. However, it still has limitations, such as crystallization or precipitation when the solubility of the drug is poor. Compared with the metered dose inhaler, it has lower dose accuracy and consistency, and higher cost.

As the most commonly used delivery system for aerosol delivery, pMDI is a pressurized system in which drugs are dissolved with excipients, such as co-solvents and surfactants, or suspended in a single propellant or mixed propellants [103]. Propellants are used to generate aerosols for inhalation. It consists of a mixture of propellants, flavors, surfactants, preservatives, and active drugs. When the drug is delivered through the pMDI, the mixture is released from the device through the metering valve and stem. Metered-dose inhalers have advantages, such as providing dose reliability, emitting accurate and consistent doses, portability, and no external power supply [104].

DPIs delivers the drug to the lung in the form of dry powders, requiring minimal coordination between the patient's breath and the device driver. DPIs do not require cold chain storage or powder reassembly, no bulky compressor or compressed air source, easy to handle, no propellant and low-cost equipment. Additionally, DPIs demonstrate suitable physicochemical stabilities, favorable flow and dispersion profiles, deep lung deposition, and improved bioavailability [105].

Clinically available inhalation formulations mainly aimed at chronic diseases such as asthma and chronic obstructive pulmonary disease (COPD) that attack rapidly and require repeated treatment over a long period of time [104]. Each therapeutic drug must be administered via a unique device, and patients need to learn how to use the corresponding device. However, using inhaler is sometimes a challenging task, which requires professional guidance and regular supervision. Otherwise, improper use may lead to ineffective treatment. For infectious diseases, antibacterial or antiviral drugs are mostly given via oral or parenteral route. Although there are different types of inhalation formulations/devices on the market, few can meet the needs such as clinical outcome, patient compliance, and economic costs. Hence, how to optimize the formulation and simplify the device remains a major challenge for the broader clinical application of inhalation formulations. For example, in patients with SARS-CoV-2 infection, administration through a metered-dose inhaler or dry powder inhaler may be negatively affected by pulmonary symptoms and comorbidities, such as limited inhalation, improper patient skills, and inability to coordinate breathing and execution [106]. For these reasons, the nebulizer may be a more practical choice for these patients, which only requires moisture breathing, and no coordinated inhalation [106]. In the following section, we will discuss the recent advances in pulmonary drug delivery systems for lung infectious disease treatment.

### Localized delivery of therapeutics against pulmonary infections

Current therapies towards lung infectious diseases mainly consist of antibiotics, antibacterial, antiviral and antifungal drugs or their combinations. Both small molecule drugs, and biological macromolecules such as peptides and proteins are involved. Conventional routes of administration mainly include oral and parenteral routes, which suffer from limitations such as insufficient therapeutic distribution at the target site and toxicities resulting from a narrow therapeutic window. Additionally, chronic infectious diseases such as TB request repeated administration over a relatively long period which results in drug resistance and poor therapeutic efficacy. Hence, strategies that can increase the therapeutic concentration at the target site and decrease systemic exposure may benefit the therapeutic outcome. Moreover, delivering drugs via pulmonary route offers a unique opportunity of repurposing drugs for the treatment of respiratory diseases. Newman SP contributed a comprehensive review on the history of drug repurposing in the treatment of respiratory diseases via pulmonary route, pointing out the critical role of repurposing as compared to new drug discovery and development [107]. In the early 1980s, inhaled antibiotics such as carbenicillin and gentamicin have been explored in the treatment of lung infections caused by *Pseudomonas aeruginosa* in patients [108]. Zanamivir, a neuraminidase inhibitor, is used to treat and prevent influenza A and B. Zanamivir (10 mg by inhalation using Diskhaler® DPI twice daily) is clinically well tolerated and can shorten the duration of symptomatic diseases [109, 110]. In a recent study, Liao et al. formulated tamibarotene into an inhalable dry powder form, and presented a promising strategy to combat various respiratory viral infections including SARS-CoV-2, influenza and MERS-CoV [111]. In terms of fungal and atypical pathogen infections, amphotericin B was injected into the cavity of the lesion to treat patients with chronic pulmonary aspergillosis (CPA) [112]. For patients at high risk of pneumocystis pneumonia (PCP), pentamidine inhalation was administered to prevent infection [113]. Since late 1990s, inhaled tobramycin has been developed for treatment of lung infections in patients with cystic fibrosis (CF) [114, 115]. With advances in inhaler devices, innovative formulations have continuously been developed, *e.g.*, tobramycin solution via a vibrating mesh nebulizer, dry powder formulation via unit dose DPI, and PulmoSphere® particles via a jet nebulizer [115–117].

In the past decade, pulmonary delivery systems based on particle technology have experienced tremendous progress. For example, liposomes, lipid NPs, polymeric NPs, polymeric micelles, and microparticles (MPs) have been explored extensively for the pulmonary delivery of various therapeutics towards lung infections. By modulating the particle size and surface properties, the deposition, retention, and cellular uptake of the particles might be improved showing enhanced specificity which could then be used for either passive or active targeted therapy of pulmonary infectious diseases [118, 119]. Generally, methods of formulation include particle formation technologies, as well as freeze-drying and spray drying technologies to produce dry powders. However, nanomaterials do have limitations, including complex formulation designs, problems of scale-up manufacturing and the difficulties to translation to actual products. Gadekar et al. contributed a nice review on the available nanomedicines for clinical interventions and pointed out the translational challenges. Due to their properties such as small size and site-specificity, nanomedicines have unique biological features, including smaller dose, reduced toxicity and improved solubility, pharmacokinetic (PK) profiles, and efficacy. Some products have entered the market with great success e.g., Doxil® and Abraxane®. Due to the complexities in formulation, future studies need to be focused on understanding the biological interactions between nanoformulations and the target, and to expedite the transition of nanomedicine from bench-to-bedside [120].

#### Liposomes and lipid NPs

The formulation of liposomal carriers and lipid NPs involve various natural or synthetic lipids such as phospholipid and its derivatives. Elhissi et al. contributed a nice chapter on liposomes for pulmonary delivery of antibacterial drugs, which summarized various liposomal formulations for pulmonary delivery of antibacterial drugs administered via pMDIs, DPIs, and nebulizers. Also, liposome encapsulated therapeutics demonstrate enhanced intracellular uptake thus likely improving the pharmacokinetic profiles of therapeutic drugs. Typically, liposomes are prepared by reverse phase evaporation techniques, which are then subjected to freeze-drying or spray drying to afford dry powders [121]. Arikace® (liposomal amikacin inhalation suspension) and Linhaliq™ (liposomal ciprofloxacin) are two products under late-stage clinical development for pulmonary application to treat lung infectious diseases [122]. Arikace® is indicated to treat *Mycobacterium avium* complex lung disease as part of a combination treatment plan for patients. Linhaliq™ developed by Aradigm is an aerosolized liposomal formulation indicated for bronchiectasis resulting from microbial infection, which displays dual-release profiles due to a mixture of liposome encapsulated and unencapsulated ciprofloxacin. The unencapsulated ciprofloxacin can release rapidly with encapsulated ciprofloxacin achieving sustained release. The intention to include a burst release trigger in the formulation design is rarely seen but could be very effective against bacterial infection. Cipolla et al. recently reported the aerosol delivery of amikacin liposomes using the eFlow® technology with the purpose to evaluate the batch-to-batch variability of the liposomal formulations with nine PARI inhalation devices, which indicates that the lipid concentration of the formulation and the device hole geometry might impact the nebulization time, but did not dramatically impact the aerosol performance parameters [123].

#### NPs

With rapid advances in material science and nanotechnology, a variety of nanoscale carriers have been studied as a means of inhalation drug delivery towards the lung. Using the solvent replacement method, clarithromycin (CLARI) was encapsulated in PLGA NPs by interfacial polymer deposition method and administered as an aerosol [124]. Compared with free CLARI, PLGA NPs loaded with CLARI were proven more effective in killing intracellular *Staphylococcus aureus* and *Mycobacterium abscess*, which may be of great potential in the treatment of patients with CF [124].

Next, the surface charge remains another critical contributor affecting the uptake of NPs by target cells. As mentioned above, the surface charge affects the interaction between NPs and the mucus, thereby delaying or preventing particles from penetrating the mucus layer [125]. The adhesion mechanism between NPs and mucus is mainly based on electrostatic/ion interactions. The negative charge of NPs can be shielded by positively charged chitosan (CS) or nonionic polyvinyl alcohol (PVA). PVA or CS are commonly used to modify the surface of PLGA NPs to improve the transport of NPs in mucus. Compared with NPs modified by PVA, NPs modified by CS displayed a higher affinity to mucus and promoted the transport of NPs in mucus, which is likely attributed to the collapse of mucus fibers to form large channels thus enhancing the penetration of NPs [126].

Besides, the surface modification of NPs with polyethylene glycol (PEG) may also improve the transport efficiency of NPs within the mucus layer. The mucus penetrating particles can be divided into two categories: (i) surface PEGylation of prefabricated NPs; (ii) self-assembled NPs containing PEG-derived polymers [127]. The dense PEG coating layer renders the NP surface neutral thus facilitating effective penetration across the airway mucus [128]. Hanes group compressed the plasmid DNA into small and highly stable NPs coated with a dense PEG layer, which showed that these PEG-coated NPs rapidly permeated through the human CF mucus layer in vitro and successfully encoded full-length plasmids of CF transmembrane conductance regulatory proteins in murine lung and airway cells [129]. In addition, Seydoux et al. intranasally instilled gold NPs (AuNPs) coated with PVA-containing positively charged (–NH_2_) or negatively charged (–COOH) polymers into mice [130]. The absorption of AuNPs by AM and dendritic cells in the trachea and bronchoalveolar lavage and pulmonary parenchyma were evaluated by flow cytometry, which showed that macrophages from broncho-alveolar lavage internalized more NH_2_-PVA AuNPs with positive charges than COOH-PVA AuNPs with negative charges [130].

Similarly, surface charge modification offers an alternative strategy for pulmonary delivery of peptides, proteins, and other biological macromolecules [131]. Zhang et al. explored a method for pulmonary protein delivery using biomimetic zwitterionic phosphorylcholine-chitosan nanoparticles (PCCs-NPs) [132]. A therapeutic protein for pulmonary fibrosis (msFGFR2c) was loaded into PCCs-NPs by ionic gelation method, and their study revealed that using PCCs-NPs as a carrier increased the survival rate of rats from 60 to 80%, which greatly improved the therapeutic effect following orotracheal administration in the bleomycin-induced pulmonary fibrosis rat model [132].

Other than the surface modification, Falciani et al. reported a nanoscale system by coupling dextran NPs with antimicrobial peptides SET-M33 (M33-NS) through non-covalent interaction, which revealed that the retention time of antimicrobial peptide SET-M33 administered through aerosol in the lungs of healthy rats was increased by three times and that the therapeutic effect was significantly improved on the BALB/c mice model of pulmonary infections [133].

#### MPs

Respirable particles, using a dry powder inhaler for drug delivery, have many advantages, such as no propellant, ease of administration and patient compatibility [134]. Therefore, pulmonary dry powders have also received increasing attentions for treating lung infections. As mentioned earlier, the best effective particle size for deep lung deposition is in the range of 1–5 μm, but this range is also within the ideal range of endocytosis of macrophages (1–3 μm) [135, 136]. For respirable powders designed to prolong local effects, phagocytosis should be avoided unless macrophages are the expected target of drug delivery. One approach is to physically camouflage the particles with polymers such as polyoxyethylene, PEG or amphiphilic phospholipid [137]. By reducing the adsorption of conditioning proteins, it provides stealthy properties for the prepared MPs. The hygroscopicity of the phospholipid-rich surface is reduced, which can further increase the proportion of respirable particles in the deep alveolar area [138].

Smyth et al. developed swellable hydrogel MPs as dry powders to achieve sustained pulmonary drug delivery. Specifically, the MPs were microencapsulated into sodium alginate semi-interpenetrating hydrogel spheres by spray drying and ion crosslinking. The dry particles were proven to have suitable aerodynamic sizes to reach the deep lung in the form of aerosols, whereas the MPs underwent swelling in the presence of tissue fluid to become larger in size to avoid rapid clearance by macrophages [139].

In addition, large porous particles (LPPs) have been designed to reach specific areas of the lung by avoid being swallowed by AMs. LPPs are characterized by large geometric size (about 10 μm in diameter), but low mass density (< 0.1 g cm^−3^) [140, 141], which display the aerodynamic characteristics of small particles (diameter of less than 5 μm). While maintaining the large size to resist the phagocytosis by AMs, the large size can also overcome the aggregation force between particles and improve aerosol performance, thus showing a favorable lung deposition profile [141, 142].

Traditional respirable MPs are often trapped in a dense mucin network and cleared by mucus. When Sharma et al. studied the treatment of TB, they discovered that barriers such as mucus and biofilm around microorganisms led to the reduction of the efficacy of antibiotics and the emergence of bacterial drug resistance. Thereby, they combined the advantages of anti-tuberculosis drugs with host defense peptides, encapsulated IDR-1018 peptides in porous PLGA microspheres modified with *N*-acetylcysteine (NAC), and developed mucus-penetrating-microparticles (NAC/PLGA-MPP), where the cationic peptide IDR-1018 demonstrated broad-spectrum activity against several infectious microorganisms, including Mtb [143]. NAC further reduced the viscosity of the mucus layer. After coating the porous PLGA-MS with NAC, the mucus penetration efficiency of the PLGA-MS particles was greatly increased. The inhaled NAC/PLGA-MPP were demonstrated to destroy the bacterial biofilm, accomplish efficient drug delivery to the lungs, resulting in a synergistic effect of anti-tuberculosis and biofilm destruction activity [144].

For Mtb treatment, Agarwal et al. loaded phage into polymeric particles to obtain phage-loaded MPs, which were deposited throughout the lungs in the form of dry powder inhalation to provide active bacteriophages for the treatment of pulmonary infection. Among them, bacteriophages infected, decomposed bacteria, and degraded biofilms, and furthermore, the mechanism of bacteria targeting has nothing to do with antibiotics, which is effective against multidrug-resistant bacteria [145–148]. The results showed that phage-carrying MPs effectively reduced *P. aeruginosa* infections and related inflammation in wild-type and cystic fibrotic catheter knockout mice and rescued mice from pneumonia-related deaths. This therapy of polymeric MPs combined with bacteriophages likely became a clinically transformable treatment option [149]. For bacteriophage therapy, Carrigy et al. reported that inhaling phage D29 aerosol before *Mtb* challenge significantly reduced the bacterial load in the mice lungs 24 h after infection, due to the fact that the mycobacteriophage could dissolve *Mtb* before macrophage uptake and granuloma formation [150].

In addition, other particles include solid lipid MPs, cyclodextrin complex MPs, and nano-embedded MPs, all of which have demonstrated their unique characteristics and pulmonary delivery potentials. For example, scientists prepared resveratrol for nano-suspensions combined with nanosuspension-in-microparticles (NS-in-MPs) and after spray drying, they found that the aerodynamic performance of NS-in-MPs significantly improved in vitro, and the lung retention time was longer than the time of resveratrol-lactose physical mixture [151].

#### Alternative delivery systems

In addition, there are alternative strategies for the localized delivery of inhaled therapeutics towards lung infections. Glycotargeting is a common strategy to achieve macrophage targeted drug delivery. The mannose receptor (CD206) on the macrophage has become a potential target of interests, which recognizes ligands with a terminal mannose, *N*-acetylglucosamine, or fucose moiety [152]. More importantly, most tissue macrophages demonstrate a high level of mannose receptor expression. Chen et al. designed a synthetic glycan copolymer of polyvalent mannose CIPX prodrug targeting AM in the form of aerosols, which showed that the targeting and enhanced internalization of the prodrug system could effectively eliminate pathogens in AM [153]. Bacterial respiratory infections are common symptoms in patients with CF or COPD, which result in mucus plugging and poor forced expiratory volume thus limiting drug penetration within the lungs [154, 155]. Antibacterial perfluorocarbon ventilation has been proposed as an alternative strategy to deliver antibiotics via pulmonary route. Specifically, Cook et al. synthesized water-in-perfluorocarbon emulsions containing aqueous antibiotics, i.e., tobramycin, which greatly improved the treatment outcome when used as an adjunct therapy to inhaled antibiotics [156].

### In vitro and in vivo models for testing therapeutics against pulmonary infections

Prior to clinical studies, relevant disease models are critical to the preliminary evaluation of drug efficacy against pulmonary infectious diseases. Typically, both in vitro cell-based and in vivo animal models have been developed to perform efficacy studies. Lehr et al. provided a comprehensive chapter on the lung disease models for testing drugs against inflammation and infection recently [157].

For establishing in vitro models, organotypic cultures mimicking the lung epithelium have been developed via differentiation through air–liquid interface cultures Also, advanced in vitro models such as organs-on-chip devices incorporate the dynamic breathing motions or fluid flow to mimic the structure and functions of the lung [158]. Besides primary cell cultures, human bronchial epithelial cells including Calu-3 and 16HBE14o—are two important cell lines widely adopted to establish pulmonary epithelium in vitro. Also, a human adenocarcinoma cell line A549 has been used to mimic primary AT-II cells using Ham’s F122 culture medium on a 25-day incubation period [159, 160]. However, these in vitro models rely on only one cell type and did not incorporate different cells to recapitulate the complex lung environment.

**Fig. 4:**
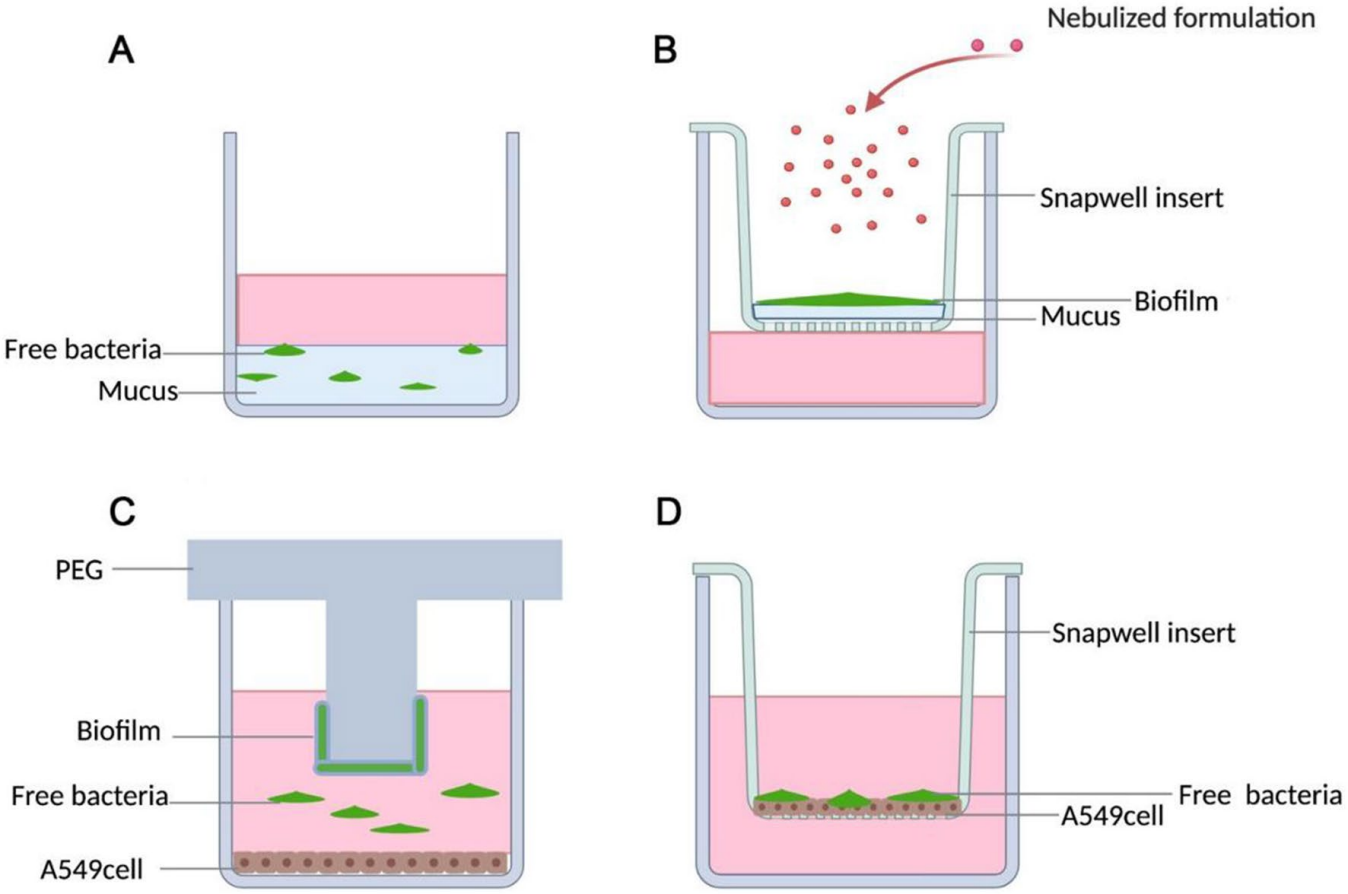
In vitro cellular infection models. Bacterial suspension was cocultured with mucus or PBS in a standard well under static conditions (**A**), air-liquid interface deposition of nebulized formulations on biofilm grown on filter inserts (**B**), biofilm on PEG inserts indirectly infecting epithelial cells (**C**), and early biofilm grown from planktonic infection of epithelial cells on filter inserts (**D**) (Figures adapted from Refs. [162–165] and created in BioRender.com)

As previously discussed, bacterial infections in the lung often lead to biofilm formation which is extremely difficult to treat. Hence, different in vitro experimental setups involving free bacteria, mucus, and epithelial cells have been generated (Fig. 4). Specifically, 100 μL of bacterial suspension was inoculated and cocultured with 100 μL of mucus or PBS in a standard well under static conditions (100% humidity, 37 °C and 0% CO_2_ for 24 h, Fig. 4A). To establish the biofilm model, *P. aeruginosa* strain was cultivated on Snapwell® inserts for 3 days to generate biofilms, which were further incubated in the presence or absence of human tracheal mucus to establish a biofilm/mucus model (Fig. 4B) [161]. The efficacy of antibiotics was then evaluated to elucidate the influence of mucus and biofilm. The mature biofilm model can further be combined with air–liquid interface cultures for the evaluation of nebulized therapies such as ciprofloxacin and mannitol [162]. Co-incubation of free bacteria with human A549 cells is used to examine the interaction between mature *P. aeruginosa* biofilms and human lung epithelial cells (Fig. 4C). The co-culture method may simulate chronic biofilm infections [163]. Co-culture of epithelial cells and free bacteria on the filter inserts simulates the early biofilm formation from planktonic infection of epithelial cells (Fig. 4D) [164].

With the ongoing pandemic, rapid advances in the development of human disease-relevant models may contribute to efficient drug screenings for finding cures against SARS-CoV-2 infection. For classic cell-based assays, cells such as Vero E6, HPAEpiC, LO2, BEAS-2B, A549, and Huh7 were infected with SARS-CoV-2 virus to afford cellular models, which could further be treated with testing compounds of varying concentrations to determine the antiviral activity [166]. Alternatively, Ebisudani et al. reported an efficient human alveolosphere based system to perform drug screenings against SARS-CoV-2 [167]. Moreover, a lung organoid model using human pluripotent stem cells (hPSC-LOs) has been generated and infected by SARS-CoV-2 to afford a relevant disease model for drug screening, which is proven effective in inducing chemokines upon viral infection similar to the responses observed in COVID-19 patients [168].

As for the animal models, mice, hamsters, ferrets, and non-human primates have been evaluated and applied in the preclinical studies of pulmonary infectious diseases. For each animal model, it is critical to recognize its unique feature, strength/weakness, as well as the differences between the model and the human disease condition. For more information, readers are suggested to refer to literatures on the specialized topic [169–171].

### Clinical trials and applications of pulmonary delivery for lung infectious diseases

Due to the unique location of pulmonary infections, inhaled formulations have been developed for the treatment of these diseases. Regarding SARS-CoV-2 pandemic, although most of the recommended drugs are given orally or intravenously, early in the course of the disease, targeted antiviral therapies may be directly administered at the site where the virus replicates, that is, the upper respiratory tract. For example, inhaled remdesivir has been extended to patients with less severe symptoms, in the early course of the disease or for reasons that are not suitable for intravenous infusion, e.g., patients with renal diseases [106]. In clinical trials, inhaled IFN-γ was demonstrated effective in the treatment of TB, but not in parenteral IFN-γ, which is likely due to the inactivation of gastrointestinal hydrolases, suggesting that aerosol therapy may effectively stimulate pulmonary macrophages [172]. As mentioned previously, in 2018, FDA accelerated the approval of Arikayce® (Amikacin liposome inhalation suspension) developed by Insmed Pharmaceuticals as part of a combined antimicrobial regimen for the treatment of adult patients with non-tuberculosis mycobacterium lung disease caused by *Mycobacterium avium* complex with limited or no treatment options. Amikacin is an aminoglycoside antibiotic with a therapeutic effect on a variety of non-tuberculous mycobacterial (NTM) lung disease, which requests intravenous administration, and its use is limited by severe ototoxicity and neurotoxicity [173]. Amikacin liposomes following direct inhalation to the lungs, ensure targeted delivery of therapeutics towards NTM-infected pulmonary macrophages [174]. Arikayce® is proven to prolong the release of amikacin in the lungs and reduce its systemic exposure, thereby reducing systemic toxicity. Following a population pharmacokinetic evaluation, compared to i.v. route of administration, inhaled formulation showed much higher concentration in sputum than in serum, which is critical to maintain high therapeutic concentration at the site of infection [175]. In addition to the above diseases, other inhaled drugs, peptides, and vaccines targeting pathogens in the lungs have also been widely used or are currently in clinical trials [176, 177].

Authors used the keywords of SARS-CoV-2 and inhalation to search registered clinical trials, which came up with over 480 trials by August 20, 2021. Herein, we briefly summarized the ongoing clinical studies based on different therapeutic modalities towards pulmonary infectious diseases (Table 1). The few approved products on the market are mainly antibacterial antibiotics indicated for pulmonary infections in the forms of either aerosols or dry powders.

**Table 1:** Inhaled formulations marketed or undergoing clinical studies for pulmonary infectious disease treatment

Disease	Formulation		Therapeutics (brand/registered name)	Development status^a^
Bacterial infection with CF	Inhalation solution		Tobramycin (Tobi®, Bramitob®)	Marketed
			Dornase alfa (Pulmozyme®)	Marketed
			Levofloxacin (Aeroquin® (formerly MP-376))	Phase III (NCT01270347, NCT01180634)
	Lyophilized powder for inhalation solution		Aztreonam lysine (Cayston®)	Marketed
			Colistimethate sodium (Promixin®)	Marketed
	Inhalable lipid particles (Pulmosphere™)		Tobramycin (Tobi® Podhaler™)	Marketed
	Inhalable dry powder		Ciprofloxacin (Cipro Inhale (BAYQ3939))	Phase III (NCT01764841)
	Excipient-free spray-dried powders		Colistimethate sodium (Colobreathe®)	Marketed
			Mannitol (Bronchitol®)	Marketed
Pulmonary TB	Aerosol	Inhalation solution	Ad5Ag85A	Phase I (NCT02337270)
	Dry powder	Inhalable dry powder	Amikacin	Phase I (NCT04249531)
Mycobacterium avium complex lung disease	Aerosol	Liposomes for nebulization	Amikacin (ARIKAYCE Kit)	Marketed
	Dry powder	Dry powder inhaler	Budesonide (PULMICORT)	Phase II (NCT04416399)
		Nanoparticle powder for inhalation	Remdesivir (GS-5734™) (VEKLURY)	Phase I (NCT04480333)
		Excipient-free dry powder	Ivermectin	Phase III (NCT04681053)
		Dry powder for nebulization	Melphalan	Phase II (NCT04380376)
		TD-0903	JAK inhibitor	Phase I (NCT04350736)
		Dry powder for nebulization	A synthetic version of Vasoactive Intestinal Polypeptide (ZYESAMI™ (aviptadil acetate))	Phase II/III (NCT04360096)
		SNG001	Interferon beta 1a	Phase II (NCT04385095) Phase III (NCT04732949)
		AP-003	Interferon alpha 2b	Phase I/II (NCT04988217)
		Dry powder inhaler	Sargramostim (Leukine®)	Phase II/III (NCT04642950)
	Aerosol	SPRAY	Ciclesonide (OMNARIS)	Phase II (NCT04381364, NCT04330586)
		HCQ01	Hydroxychloroquine sulfate	Phase I/II (NCT04731051)
		S-1226 (8%)	Carbon dioxide (8%) and perflubron (PFOB)	Phase II (NCT04949386)
		Inhalation Solution	ILOPROST (VENTAVIS)	Phase II (NCT04445246)
		Nebulizer inhalation solution	Saline containing 0.3% hyaluronic acid sodium salt (Yabro®)	Phase II (NCT04830020)
		Nasal spray	Ivermectin	Phase II (NCT04510233)
		Nasal spray	Sodium Pyruvate	Phase II/III (NCT04824365)
		Inhalation solution	Adenosine	Phase II (NCT04588441)
		Inhalation solution	13-cis retinoic acid	Phase II (NCT04396067)
		Inhalation solution	Virazole (Virazole®)	Phase I (NCT04551768)
		Inhalation solution	Nitric oxide (RESP301)	Phase II (NCT04858451)
		Inhalation solution	Furosemide	Phase II/III (NCT04588792)
		Nebulized inhalation solution	Captopril	Phase II (NCT04355429)
		Inhalation solution	synthetic form of Human Vasoactive Intestinal Polypeptide (Aviptadil)	Phase II (NCT04536350)
		Nebulized inhalation solution	Alpha 1-Antitrypsin (GlASSIA)	Phase I (NCT04385836)
		Nebulized inhalation solution	Interferon beta 1b (EXTAVIA)	Phase II (NCT04469491)
		Nebulizer inhalation solution	GM-CSF (rHuGM-CSF) (Molgramostim)	Phase II (NCT04569877)
		Nebulized inhalation solution	Sargramostim (GM-CSF) (Leukine®)	Phase II (NCT04707664)
		BI 767551	Antibody against the coronavirus SARS-CoV-2	Phase II/III (NCT0489447, NCT04822701)
		DZIF-10c	SARS-CoV-2-neutralizing monoclonal antibody	Phase I/II (NCT04631705)
		DAS181	Recombinant Sialidase Protein	Phase II/III (NCT04354389)
		Inhalation Solution	Dornase Alfa (PULMOZYME)	Phase III (NCT04402970)
		Inhalation solution	The low molecular weight filtrate of human serum albumin (Ampion)	Phase II (NCT04868890)
		Inhalation solution	A novel recombinant antiviral protein (Novaferon)	Phase III (NCT04669015)
		Powder for inhalation solution	Recombinant tissue-Plasminogen Activator (rt-PA)	Phase II (NCT04356833)
		Nebulizer inhalation solution	Ad5-nCoV (Recombinant Novel Coronavirus Vaccine)	Phase I (NCT04552366) Phase I/II (NCT04840992)
		Natural nano-sized vesicles for inhalation	EXO1 EXO2	Phase II (NCT04491240, NCT04602442)
		Overexpressing CD24 nano-sized vesicles for inhalation	EXO-CD24	Phase I (NCT04747574)
		COVID-19 specific T cell derived nano-sized vesicles for inhalation	CSTC-Exo	Phase I (NCT04389385)
		Nano-sized vesicles for inhalation	MSCs-derived exosomes	Phase II (NCT04445246)
		Inhalation solution	PUL-042	Phase II (NCT04312997, NCT04313023)

^a^Further information on the status of clinical trials can be found at http://clinicaltrials.gov

^b^Some information was retrieved from references [176, 177]

SARS-CoV-2 infection induces severe lung inflammation and injury. When infected by SARS-COV-2, the immune cells will be activated at a high level and produce a large number of inflammatory cytokines and chemical mediators. However, when the immune system is over activated, the excessive inflammation and immune response will occur, which is called cytokine storm. A variety of factors may induce cytokine storm including viruses, bacterial components, superantigens, toxins, and chimeric antigen receptor T cells. Early control of the cytokine storm is essential to improve the survival rate of COVID-19 patients [178, 179]. At present, there is no specific treatment for the infection-induced cytokine storm in the clinical practice, and non-specific combination therapies are often used such as anti-infective drugs, glucocorticoids, nutritional support, and artificial ventilation assistance. A recent study by Ali and El-Mallakh report that inhaled lidocaine is expected to reduce inflammatory cytokines, and represents a viable adjuvant therapy for COVID-19 patients with cytokine storms [180]. Inhalation administration offers an alternative option for the management of SARS-CoV-2 induced pulmonary symptoms. The pulmonary inhalation formulations may reach the deep lung directly and rapidly. Importantly, the therapeutic drugs should have a fast onset of action and be easy to use. Two types of pulmonary inhalation preparations are used for the treatment of SARS-CoV-2, i.e., dry powders and aerosols. Specifically, the inhalation solution needs to be administered via a nebulizer, and dry powders often need to be suspended in physiological saline for inhalation. The therapeutics used for inhalation are mainly divided into small molecule and macromolecule drugs. Small molecule drugs include glucocorticoids, and antiviral drugs, e.g., budesonide, ciclesonide and remdesivir. A recent study by Ramakrishnan et al. reported that early inhalation of budesonide therapy may reduce the possibility of emergency medical treatment [181]. Juan Pimentel et al. reviewed the clinical studies of remdesivir in the management of SARS-CoV-2 based on 23 registered trials on over 30,000 participants, which concluded that remdesivir following intravenous injection might shorten the time to clinical improvement among hospitalized adults with severe COVID-19 [182]. However, inhaled remdesivir study (NCT04539262) is currently under Phase I clinical trials with no public results available yet. Further work is needed to confirm the efficacy of inhaled remdesivir. Macromolecular therapeutics via pulmonary inhalation including interferon β1β, interferon β1α, and interferon α2β are under Phase I/II clinical trials. Yu et al. reported that interferon α2β aerosol inhalation therapy following a twice daily plan might contribute to improved clinical outcomes in patients with SARS-CoV-2 infection [183, 184]. Based upon a dual role of anti-viral and immune modulation, neutralizing antibodies such as DZIF-10c (BI 767551) are generated against SARS-CoV-2. DZIF-10c is an antibody derived from recovered COVID-19 patients under Phase I/II clinical trials, and the trial result has not yet been published [185]. Therapeutic exosomes for the treatment of SARS-CoV-2 include EXO1, EXO2, MSC-Exo and EXO-CD24. Akbari and Rezaie reported that MSC-Exo represents a promising therapy against SARS-CoV-2 infection, while further work needs to be performed to confirm its efficacy clinically. Specifically, EXO-CD24 is proven to suppress the “immune storm” in patients with SARS-CoV-2 infection with a great therapeutic potential against SARS-CoV-2. In the Phase I clinical trial, EXO-CD24 substance was administered to 30 patients whose conditions were moderate or worse, and all 30 recovered with 29 of them recovering within 3 to 5 days [186]. Moreover, the recombinant novel coronavirus vaccine from CanSinoBIO (Ad5-nCoV) is currently the only inhalation vaccine that has entered the clinical trial [184]. Compared with injected vaccines, inhaled vaccines have the advantages of small doses, fast onset of action, low cost and mucosal immunity. Per results from a Phase I clinical trial, the two-doses of the inhaled Ad5-nCoV vaccine triggered a similar immune response to the single-dose of the injected version without causing serious side effects [184].

## Conclusions and outlook

Due to the air–liquid interface, lung associated diseases are characterized by a complex microenvironment which presents great challenges towards efficient drug delivery and effective therapeutic outcome. Regarding pulmonary infectious diseases, the scientific community suffers from a lack of basic knowledge towards disease etiology and pathology. Hence, one goal of this study is to put together known data and studies. Specifically, this review discusses pulmonary delivery strategies towards lung infections, which we believe offers an alternative perspective besides systemic or oral routes of administration.

Considering the major global health crisis in the past century, most have been related with respiratory infections. The highly efficient COVID-19 vaccine development is partly due to the cumulative experience with mRNA therapeutics and lipid nanotechnology. Thus, it is necessary to reserve relevant technologies through a systematic review. Regarding pulmonary infections, it is critical to identify the differences between pathogens such as bacteria and viruses, and the acute/chronic stage of infections when considering the design of formulations. For the mode of inhaled drug delivery in the lung, although the natural and physiological barriers in the lung will inevitably have a negative impact on drug delivery, with the advances of materials, the use of nanotechnology and surface modifications can help overcome the biological barriers to achieve efficient drug delivery towards the lung lesions with the benefits of no first-pass effect, rapid absorption, and improved bioavailability. Despite the improved drug targeting index and enhanced distribution towards the lung, the physicochemical properties of the particulate delivery systems such as size, shape and charge, and how these properties may impact the distribution profile need to be elucidated. On the other hand, carrier toxicity remains a potential issue to be carefully addressed. The surface charge, chemical composition, and excipients used in the formulation or the delivery system may affect the normal function of the lungs, leading to problems, such as pulmonary inflammation, fibrosis, and lung cancer.

Localized delivery via inhalation offers a non-invasive route of administration. However, a gap remains between evaluation models and clinical application. Some drugs or therapies that are proven effective in animals, turn out ineffective in clinical trials. Hence, future studies shall focus on disease-relevant animal models and highly efficient evaluation platforms to assess the efficacy and the safety of novel inhalation therapies to achieve successful clinical translation. Finally, although an increasing number of inhalation formulations have been developed, only a small number of therapeutics can be used for clinical translational studies. Hence, attentions should also be focused on the critical factors that affect the successful inhalation of drugs, and finding ways to improve the stability and uniformity of formulations so that more drugs can be used for inhalation.

Overall, with the continuous development of material science and targeting strategies, there will be more and more DDS and carriers available to achieve highly efficient therapy of pulmonary infections. Meanwhile, efforts need to be contributed to resolve the unmet needs to promote clinical translational studies.

## Data Availability

Not applicable.

## Abbreviations

AT-II: Alveolar type II
ACE2: Angiotensin-converting enzyme 2
AMs: Alveolar macrophages
RBD: Receptor binding domain
COPD: Chronic obstructive pulmonary disease
CF: Cystic fibrosis
CLARI: Clarithromycin
CS: Chitosan
DDS: Drug delivery systems
DPIs: Dry powder inhalers
FcR: Fc receptors
LPS: Lipopolysaccharide
LPPs: Large porous particles
Mtb: *Mycobacterium tuberculosis*
MPs: Microparticles
NPs: Nanoparticles
NAC: *N*-Acetylcysteine
NS-in-MPs: Nanosuspension-in-microparticles
NTM: Non-tuberculous mycobacterial
PCL: Periciliary layer
PS: Pulmonary surfactant
pMDIs: Pressurized metered-dose inhalers
PVA: Polyvinyl alcohol
PEG: Polyethylene glycol
PCCs-NPs: Phosphorylcholine-chitosan nanoparticles
TB: Tuberculosis
MERS-CoV: Middle East respiratory syndrome virus
CPA: Chronic pulmonary aspergillosis
PCP: Pneumocystis pneumonia
